# Photoplethysmographic Time-Domain Heart Rate Measurement Algorithm for Resource-Constrained Wearable Devices and its Implementation

**DOI:** 10.3390/s20061783

**Published:** 2020-03-23

**Authors:** Marek Wójcikowski, Bogdan Pankiewicz

**Affiliations:** Faculty of Electronics, Telecommunications and Informatics, Gdańsk University of Technology, 80-233 Gdańsk, Poland; bpa@eti.pg.edu.pl

**Keywords:** heart rate, photoplethysmography, PPG, time-domain, wearable device

## Abstract

This paper presents an algorithm for the measurement of the human heart rate, using photoplethysmography (PPG), i.e., the detection of the light at the skin surface. The signal from the PPG sensor is processed in time-domain; the peaks in the preprocessed and conditioned PPG waveform are detected by using a peak detection algorithm to find the heart rate in real time. Apart from the PPG sensor, the accelerometer is also used to detect body movement and to indicate the moments in time, for which the PPG waveform can be unreliable. This paper describes in detail the signal conditioning path and the modified algorithm, and it also gives an example of implementation in a resource-constrained wrist-wearable device. The algorithm was evaluated by using the publicly available PPG-DaLia dataset containing samples collected during real-life activities with a PPG sensor and accelerometer and with an ECG signal as ground truth. The quality of the results is comparable to the other algorithms from the literature, while the required hardware resources are lower, which can be significant for wearable applications.

## 1. Introduction

Advancements in modern technologies enabled field monitoring of some parameters of human health [1]; for example, heart monitoring is used for off-hospital monitoring and in fitness and professional sport activities. The most commonly measured value is the heart rate (HR), although advanced applications also use other values, e.g., pulse irregularity, as well as biometric identification or analysis of accurate electrical signals that cause heart contraction, i.e., electrocardiography (ECG) [2]. Accurate ECG requires connecting electrodes to the patient’s body in several different places, which is inconvenient for the patient, and it can be used only in certain situations. A much more convenient method is measuring the pulse on the wrist by using photoelectric methods. The skin of the wrist is irradiated with single or multicolor light, and then the reflected light is measured. The intensity of the reflected light depends on the absorption of the skin, which depends on the blood volume supplied to the tissues. In this way, the received signal contains information about the current blood supply to the vessels near the measuring device. This method, introduced by Hertzman [3], is known as photoplethysmography (PPG). Unfortunately, PPG signals obtained from a moving person’s wrist are weak, distorted, and contain noise. The noise level is often higher than a usable PPG signal. Correct analysis of a low-quality PPG signal is a very challenging task and can consume significant processing time, energy, and resources.

Heart rate estimation from wrist PPG is now a popular area of investigation, and many algorithms for such a task have been proposed [4]. Some of them require significant computing power and memory usage, blocking their application in small portable low-power devices. There are two main approaches used: time-domain sophisticated filtering and frequency-domain processing. Both are often accompanied by a movement sensor (accelerometer) for movement-based artefacts/spectrum removal.

A straightforward approach toward heart rate estimation is peak detection in a periodic signal. One of the simplest possibilities is to use threshold or auto-threshold values in the signal time window [5,6]. Another way is to use transforms such as continuous wavelet [7] or Hilbert [8]. In [9], a nonlinear filter bank was used, with variable cutoff frequencies. In [10], the detection algorithm was based on a time-varying autoregressive model, with a Kalman filter used for autoregressive parameter estimations. Hidden Markov models were used in [11] for combining structural and statistical knowledge of the signal in a single parametric model. A neural network adaptive whitening filter to model the lower frequencies of the signal is presented in [12]. Much recent work is concentrated on methods using time-frequency spectra [13,14] and deep-learning approaches [15,16,17]. A review of the current state-of-the-art signal-processing techniques for HR estimation from a wrist PPG signal can be found, for example, in [4,18].

The proposed solution of time-domain heart rate measurement algorithm (TDHR) consists of three main blocks: signal conditioning, peak detection, and heart-rate-measuring blocks. In this work, the modified Automatic Multiscale-based Peak Detection (AMPD) algorithm from [19], together with a bandpass filter/limiter, was used for finding the HR from a wrist-based PPG signal. All of the signal processing was done while taking into account the need for low power, low resources, and computing power utilization, necessary for a self-sufficient mobile sensor. The main contributions of this paper are as follows:

- A two-stage input-signal-conditioning digital nonlinear filtering block with limiter;
- Application of the AMPD algorithm for HR peak detection;
- Modification of the AMPD algorithm toward efficient implementation in low-power resource-constrained hardware;
- The proposition of a time-domain heart-rate-measuring algorithm with an accelerometer-based false measurement removal.

This paper is organized as follows. In Section 2, the signal conditioning block is described, which is followed by the peak detection algorithm described in Section 3 and the heart rate calculation block presented in Section 4. The proposed algorithm was evaluated by using a multi-hour dataset; the results of the evaluation and the comparison to the other solutions from the literature are presented in Section 5. Section 6 contains the details of the implementation in a low-power wearable device. Finally, conclusions are provided in Section 7.

## 2. Signal Conditioning

**Figure 1:**
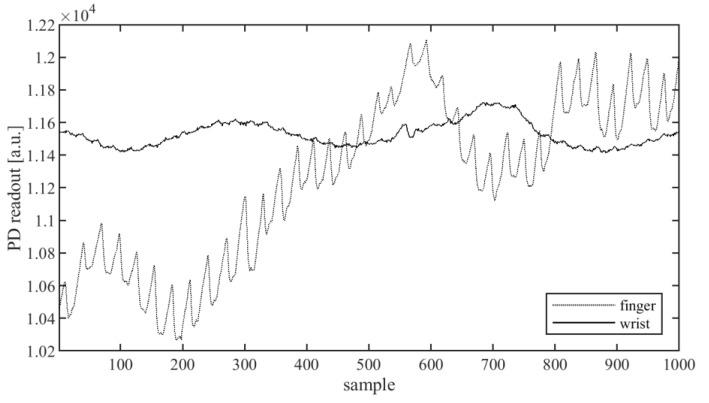
Photodetector(PD) waveforms: raw signals obtained from photoplethysmography (PPG) sensor in reflective configuration placed on the index tip (dotted line) and on the wrist (continuous line).

PPG-signal measuring is usually done with an infrared (IR), red, or green light-emitting diode (LED) as the light source and a photodetector (PD) receiving the reflected or transmitted light. Often, instead of one, a few LEDs and/or PDs are used, providing the possibility to choose the best observed signal or to use additional preprocessing, such as, for example, averaging. Two measure modes can be used: reflectance and transmission. In the first case, the LED and PD are placed on the same skin surface, close to each other; the typical spacing between the PD and LED is in the range of 5–15 mm. The PD measures the light reflected from the tissue. In transmission mode, the LED and PD are placed on the opposite sides of a human body part, and the light reaching the PD goes through the whole body part. The commonly used places on a human body for such PPG devices are the fingertip, wrist, earlobe, forehead, torso, ankle, and nose [18,20]. The wrist is the most convenient place to mount the monitoring device for everyday life, but this is not optimal regarding the strength of the signal. Due to the relatively large thickness and the presence of bones, only the reflectance method of measurement is practical on the wrist. In Figure 1, the waveforms of the signal received from the sensor in reflectance mode on the index finger and on the outer wrist, where a watch is usually worn, are presented. A useful signal carrying information about the pulse is present in the form of the peaks with the period of about 30–40 samples superimposed on the curve. As shown in Figure 1, the amplitude of these peaks for the signal measured at the finger is about 200, while the amplitude measured at the wrist is much lower, about 30–50, while the average signal level (baseline) is about 11,100.

**Figure 2:**
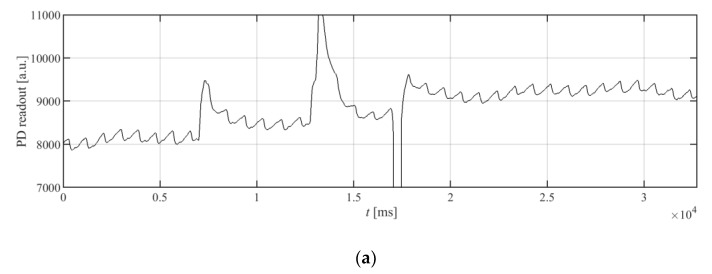

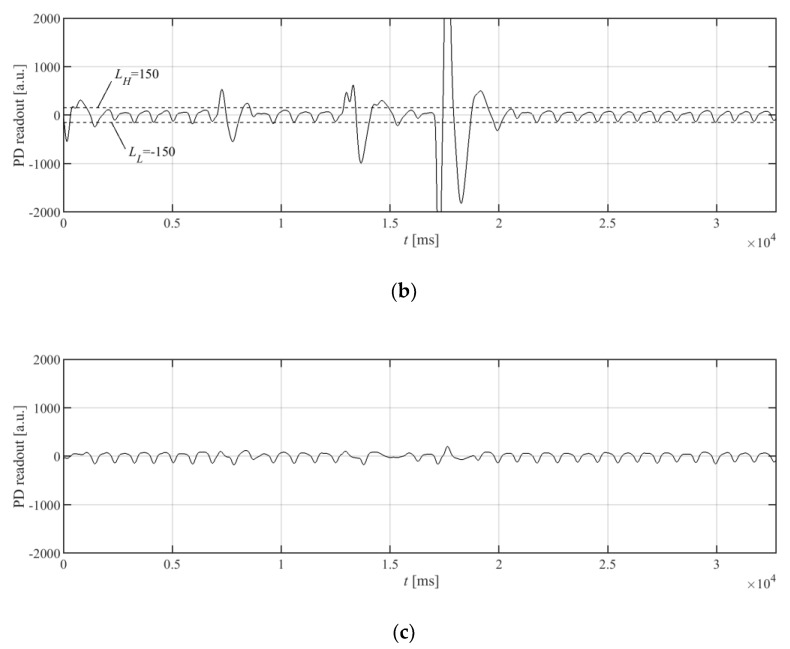
Waveforms of the signals from the PPG detector: (**a**) the raw signal; (**b**) the raw signal from (**a**) filtered only by a band-pass filter; and (**c**) the raw signal from (**a**) filtered first with the limiting section described in the paper and then by the same biquadratic filter as used in (**b**).

As can be seen from the waveforms from Figure 1, the signal from the wrist is much weaker; moreover, each signal has a variable offset, and it contains noise and interference. To extract the heart rate in a time-domain, the signal must be preprocessed, so that the peak detection algorithm can find the peaks. The simplest approach is to use a band-pass filter that can help to reduce the noise and eliminate the constant component of the signal. Such a solution is in common use [4]. However, the signal at the input of the filter can have significant changes of the constant component, as well as signal fluctuations caused by wrist movement, resulting in large peaks and oscillations at the output of the band-pass filter, as can be seen in Figure 2a,b.

**Figure 3:**
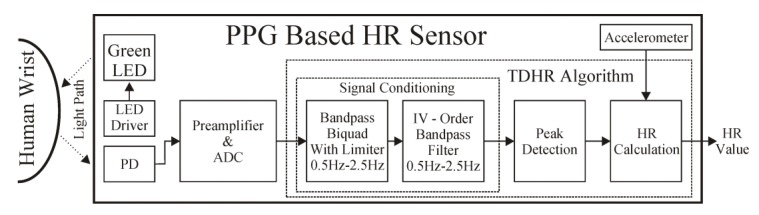
Block diagram of PPG signal acquisition, preprocessing, and final HR estimation by TDHR algorithm.

The proposed approach for PPG signal acquisition and processing is presented in Figure 3. The raw signal obtained from the PD is fed, in the first step, to the band-pass biquad section, with an internal limiter. The output value of the standard biquad section using direct version I is given by Equation (1):

(1) $$y_{i}=\frac{1}{a_{0}}\left(b_{0}x_{i}+b_{1}x_{i-1}+b_{2}x_{i-2}-a_{1}y_{i-1}-a_{2}y_{i-2}\right),$$

where *y_i_* is *i*-th output sample; *x_i_* is *i*-th input sample; *a*_0_, *a*_1_, and *a*_2_ and *b*_0_, *b*_1_, and *b*_2_ are, respectively, the denominator and nominator coefficients of the biquad transfer function. The biquad section with the internal limiter works in two steps: First, it calculates candidate *y_C,i_* for the output value, according to Equation (1), and then it uses Equation (2) to update the actual output value:

(2) $$y_{i}=\left\{ \begin{array}{l} y_{C,i}\;\mathrm{if}\;L_{L}\leq y_{C,i}\leq L_{H}\\ L_{L}\;\mathrm{if}\;y_{C,i}<L_{L}\\ L_{H}\;\mathrm{if}\;y_{C,i}>L_{H} \end{array}\right.,$$

where *L_L_* and *L_H_* are the limiter’s parameters. These parameters should be selected so that the PPG signal from the heart contractions will remain intact, while distortions resulting from hand movements should be cut off. Figure 2a shows the PPG signal containing distortions taken from a wrist. For this case, the limiter parameters should therefore cut off these high distortions, while the small sawtooth waveform should not be affected. The signal after the bandpass filter does not contain a constant component, as is presented in Figure 2b. In the case of the signal from Figure 2b, good choices for the *L_L_* and *L_H_* parameters’ values could be −150 and 150, respectively. Figure 2c shows the results of preprocessing for such a limiter, together with the band-pass biquad section. In general, these values for a given PPG system should be selected as about 100–150% of the negative and positive amplitude of the useful signal, respectively.

In the second stage of the proposed preprocessing block, a typical fourth-order band-pass filter built from two biquads was employed. The band-pass of both stages was set to 0.5–2.5 Hz.

The introduction of the limiting section in the form of a single biquad stage significantly reduces the rapid changes of the signal at the input of the band-pass filter and improves its recovery after a rapid change of the constant component in the input signal. An example of distorted signal, together with the accelerometer readouts, is presented later in this paper.

## 3. Peak Detection

The conditioned signal from the detector was used as an input to the block responsible for finding the peak values of the signal; the heart rate can then be easily calculated from the detected peaks. The detection of peaks is based on the AMPD algorithm [19]. The AMPD algorithm has the capability to work with noisy periodic and quasi-periodic signals. It needs the input signal to be linearly detrended, but the use of the input filter of band-pass characteristic with the limiter described in the previous section satisfies this requirement. The AMPD algorithm performs well for the filtered PPG signal, but it is computationally expensive, which can be unacceptable for wearable devices. The need to calculate a large matrix with real-valued elements, where moving windows are used, can be avoided due to the modifications of the algorithm proposed in further parts of this paper. This section starts with the detailed description of the AMPD algorithm, and then the proposed modifications are introduced. In this way, the authors wanted to make it easier for the reader to track changes that were applied to the original algorithm, without having to refer to the reference.

The main part of the AMPD algorithm is the Local Maxima Scalogram (LMS) matrix M of elements, *m_k,i_*, which is calculated for the discrete uniformly sampled signal *x* = [*x*_1_, *x*_2_, …, *x_N_*] in the analyzed window, where *N* is the constant number of samples, and scale *k* defines the moving window of varying length, *w_k_*, according to Equation (3):

(3) $$m_{k,i}=\{\begin{array}{cc} 0 & x_{i-1}>x_{i-k-1}\wedge x_{i-1}>x_{i+k-1}\\ r+1 & \mathrm{otherwise} \end{array},$$

where *w_k_* = 2*k* | *k* = 1,2, …, *L*, *k* is *k*-th scale of the signal, *L* = ceil(*N*/2) − 1, and *r* is a uniformly distributed random number of values [0,1]. The values of *m_k,i_* are calculated for every scale *k* and *i* = *k* + 2, *k* + 3, …, *N* − *k* + 1.

Having calculated the LMS matrix, the next step in the AMPD algorithm is to calculate the scale-dependent distribution of zeros in the LMS, by calculating vector *γ* = [*γ*_1_, *γ*_2_, …, *γ_k_*]:

(4) $$\gamma_{k}=\sum_{i=1}^{N}m_{k,i}\;\mathrm{for}\;k\in \;\{1,\;2,\ldots ,\;L\}$$

and the global minimum of *γ*, *λ* = arg min(*γ_k_*). The value of *γ* is used to obtain the matrix M_r_, which is the matrix M with deleted bottom rows for *k* > *λ*. The peaks are then found for indexes, *i*, for which the column-wise standard deviation, *σ_i_*, is equal to zero:

(5) $$\sigma_{i}=\frac{1}{\lambda -1}\sum_{k=1}^{\lambda }{\left[{\left(m_{k,i}-\frac{1}{\lambda }\sum_{k=1}^{\lambda }m_{k,i}\right)}^{2}\right]}^{\frac{1}{2}}.$$

Parameter *λ* enables the determination of how many rows of matrix M should be used for calculating the matrix M_r_. The noisier the signal from the PPG sensor, the larger the value of *λ*.

In this paper, the authors propose modifying the AMPD algorithm toward a more efficient implementation. From practical observation, it has been inferred that the signal is too noisy, and it is of no use for peak detection and heart rate calculation, when we have the following:

*λ* > *λ*_max_ (6)

The value of *λ*_max_ = 17 was found empirically to be a good choice. Therefore, it is practical to assume a priori, that the signals for which the condition (6) is true are of no use and the full matrix M is not used. To save the memory, only the matrix M_r_, instead of M, can be calculated and stored together with the vector *γ*.

Processing and storing floating point values, *m_k,i_*, requires storage space and processing resources. To simplify the processing, the authors propose replacing real-valued elements of matrix M_r_ with the matrix M_r_’ containing 1-bit binary values, *m*’*_k,i_*, as follows:

(7) $${m^{\prime }}_{k,i}=\{\begin{array}{cc} 0 & x_{i-1}>x_{i-k-1}\wedge x_{i-1}>x_{i+k-1}\\ 1 & \mathrm{otherwise} \end{array}.$$

This saves a lot of the device’s memory, requires only integer operations, and results in another simplification: Instead of calculating the column-wise standard deviation *σ_i_* from Equation (5), calculating the column-wise summation presented in Equation (8) can be used:

(8) $$s_{i}=\sum_{k=1}^{\lambda }{m^{\prime }}_{k,i}.$$

**Figure 4:**
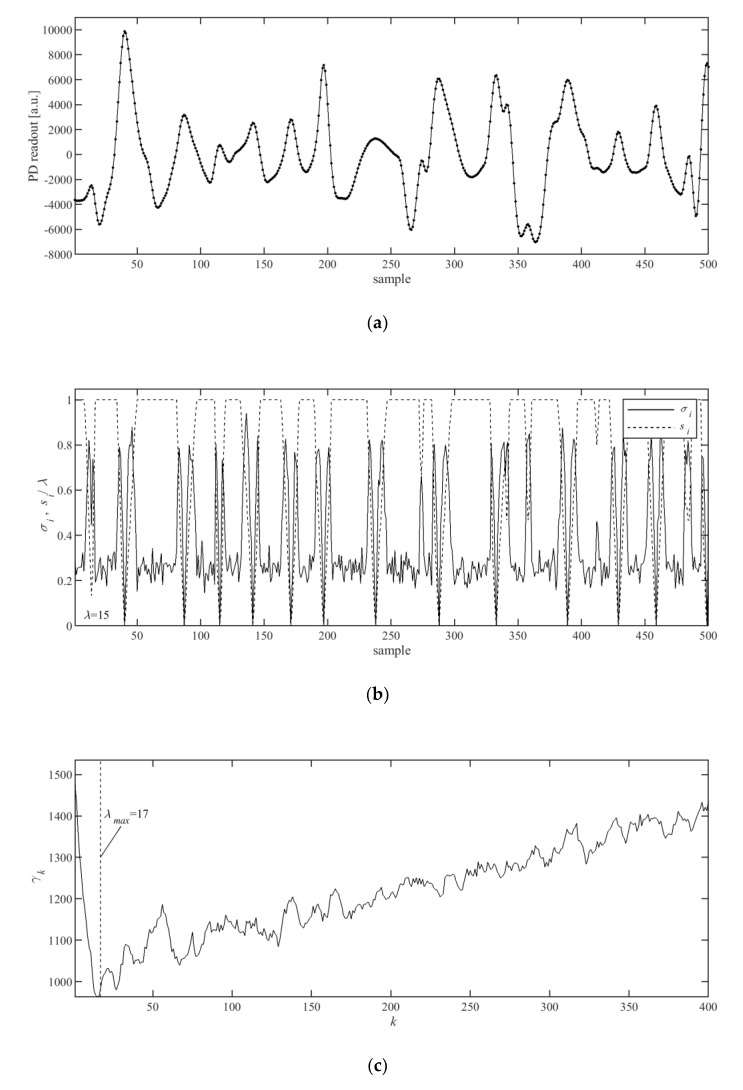
Sample of (**a**) filtered input PPG signal, (**b**) calculated and normalized values of *σ_i_* from Equation (5) and *s_i_* from Equation (8), and (**c**) calculated values of *γ_k_* from Equation (4).

As the *m*’*_k,i_* are 1-bit binary values, *s_i_* can be calculated with fast integer summation. The indices of peaks *p_i_* can be located by finding all indices, *i*, for which *s_i_* = 0. The values of *σ_i_* and *s_i_*, together with the values of *γ_k_* were shown in Figure 4b,c, for an exemplar input signal (Figure 4a).

**Figure 5:**
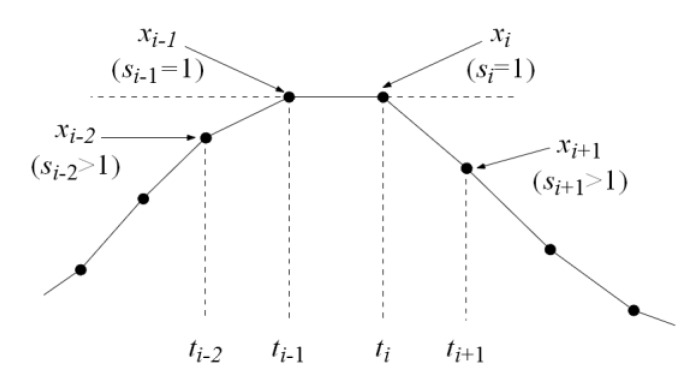
An example of special case of a “flat” peak consisting of two equal values at *t_i_*_-1_ and *t_i_*.

The PPG signal from a wrist is weak, as can be seen in Figure 2c, and when sampled, the situation of a “flat” peak with two equal values for samples *t_i_*_-1_ and *t_i_*, as shown in an example in Figure 5, can sometimes occur. Such a peak would neither be detected by the AMPD algorithm nor its modified version proposed in this paper. To improve the peak detection in such a case, an additional rule was introduced: The peak is also detected at time *t*’*_i_*.

(9) $${t^{\prime }}_{i}=\frac{t_{i-1}+t_{i}}{2}$$

for which the following simple condition (10) holds for *s_i_* from Equation (8):

*s_i_*_-2_ > 1 ∧ *s_i_*_-1_ = 1 ∧ *s_i_*=1∧ *s_i_*_+1_ > 1. (10)

**Figure 6:**
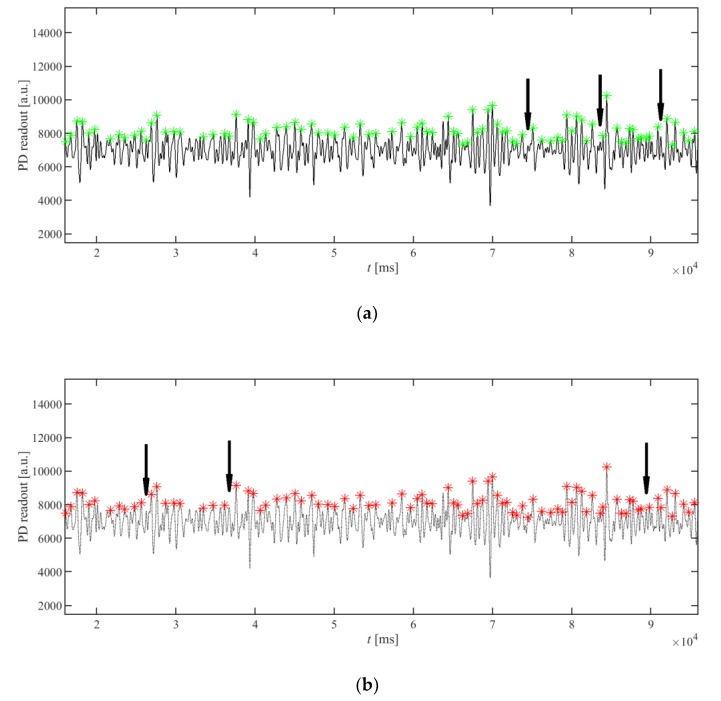
Comparison of the results of the peak detection algorithm proposed in this paper (**a**) with the AMPD peak detection algorithm (**b**) from [19] on the same waveform. The arrows indicate the peaks not detected by one of the algorithms but detected by the other one. All other peaks were detected at the same positions, by both methods.

The proposed simplified peak detection algorithm was compared to the original AMPD algorithm presented in [19]. For this purpose, the PPG signals from the PPG-DaLia database [15] were used, and the detected peaks were compared. As the original AMPD peak detection algorithm uses a random variable to fill the LMS matrix, its results are slightly different from run to run, depending on the seed value of the random number generator. The results are very similar, while the modified version can be implemented more efficiently. An example is presented in Figure 6: the arrows indicate the missing peaks, which were exclusively detected by the other algorithm. It can be seen that only a few peaks are differently detected.

The presented example uses an unfiltered PPG signal to present the robustness of the algorithm. The signal after filtration would be more regular; thus, the differences in the results between the two versions of the algorithm would be even smaller.

## 4. Heart Rate Calculation

**Figure 7:**
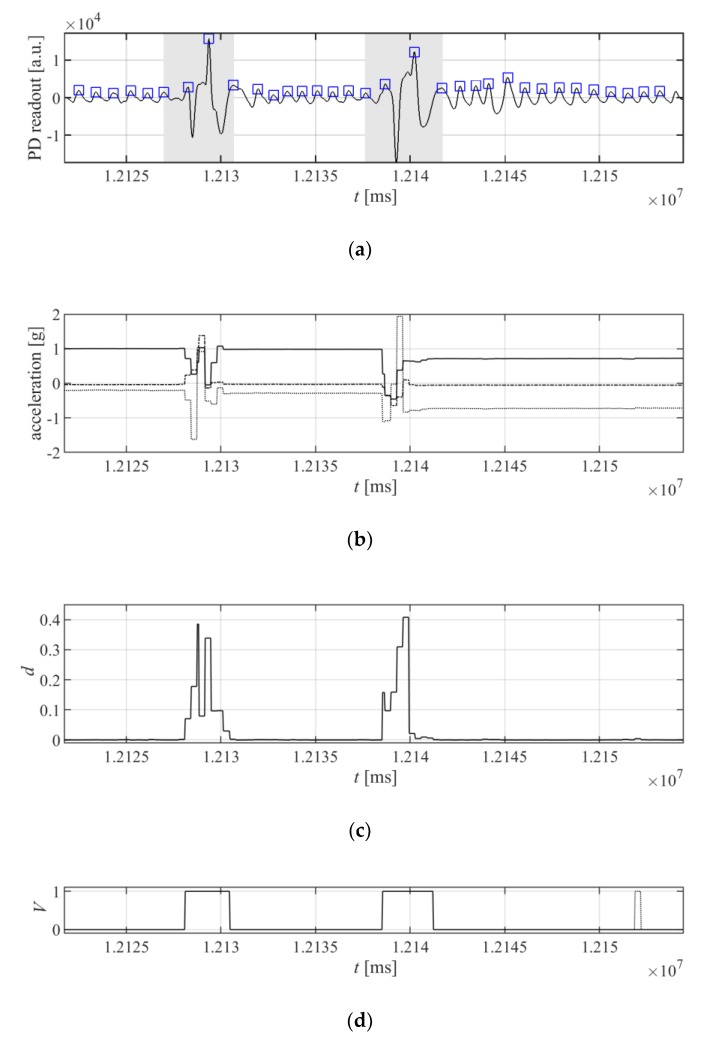
Elimination of the patient’s movement from pulse-signal detection: (**a**) the PPG signal distorted with the patient’s movement; the areas in gray are excluded from pulse period calculation due to the detected movement; (**b**) acceleration values read from the accelerometer placed together with the heart rate detector on the wrist, (**c**) and the values of movement *d_i_* from Equation (11); (**d**) signal *d_i_* after threshold, as in Equation (12), and with the eliminated short-term peaks (the eliminated peak is shown with a dotted line).

In an ideal situation, where the patient is not moving, the detected peaks from the peak detection algorithm can be directly used to calculate the heart rate. However, the PPG signal can be seriously distorted by the movement of the patient. Each movement of the patient can cause a change in the saturation of the tissues with blood, and a sensor displacement on the wrist, which results in artefacts in the signal received by the optical detector. To mitigate this problem, the accelerometer is used for detecting the movement of the patient’s hand. The movement data are aligned with the detected peaks, and the periods between the peaks which are affected by movements are discarded from the calculation of the pulse period. The example of the elimination of the patient’s movement is presented in Figure 7.

To find the time periods that will be excluded from period calculations, first the acceleration values from the accelerometer are differenced to obtain the movement indicator, *d_i_,* according to the following equation:

(11) $$d_{i}=\frac{1}{G}\sqrt{{\left(g_{x,i}-g_{x,i-1}\right)}^{2}+{\left(g_{y,i}-g_{y,i-1}\right)}^{2}+{\left(g_{z,i}-g_{z,i-1}\right)}^{2}},$$

where *i* is the index of the sample; *g_x,i_*, *g_y,i_*, and *g_z,i_* are the acceleration values read from the accelerometer for axes X, Y, and Z, respectively; and *G* is a constant value used for normalization to obtain *d_i_* ∈ [0,1] for all *i* and for the acceleration values in *g_x,i_*, *g_y,i_*, and *g_z,i_* in the accelerometer’s full measuring range. The movement values, *d_i_*, are then compared to the constant, *H*, to obtain a digital binary signal *V_i_*:

(12) $$V_{i}=\{\begin{array}{cc} 1 & d_{i}>H\\ 0 & \mathrm{otherwise} \end{array}$$

Signal *V_i_* is then filtered in time, to eliminate spikes longer than *T_s_*. The values of *H* and *T_s_* were experimentally set to *H* = 0.0025 and *T_s_* = 500 ms for the accelerometer range ±2 g.

For the final result of the heart rate, the inverse of the median of the periods between the peaks not affected by the movement is calculated. For a valid result, a minimal number of peaks, *P*, is required, and it is calculated as shown in Equation (13):

(13) $$P=floor\left(\frac{N\cdot BPM_{\min}}{60f_{s}}\right),$$

where *f_s_* is the sampling frequency, and *BPM*_min_ is the minimal heart rate required to be measured by the system.

## 5. Evaluation on Dataset

For the purpose of evaluating the algorithm, the PPG-DaLia database [15], containing more than 35 h of data recorded from 15 persons, was used. The database contains signals collected from a PPG sensor, accelerometer, and ECG, where the ECG is used as ground truth. As described in [15], ground truth heart rate values were obtained from an ECG signal processed by R-peak detection algorithm [21]. Then, the detection results were manually inspected and corrected, mainly for a few cases where significant motion was observed. The ECG signal was segmented with a shifted window; ground truth heart rate was finally calculated as the mean heart rate within each window. The signals were collected during eight different types of typical daily life activities, under controlled but close-to-real-life conditions. This dataset is the longest one available to the authors. The accuracy of the algorithm was evaluated by using the mean absolute error (*MAE*) metric of beats per minute (bpm), calculated by using the sliding window approach of length 8 s, with a 2 s shift, according to the following equation:

(14) $$MAE=\frac{1}{W}\sum_{j=1}^{W}\left(BPM_{est}(j)-BPM_{ref}(j)\right),$$

where *W* is the total number of windows, *BPM_est_*(*w*) is the heart rate in bpm for window *j*, and *BPM_ref_*(*j*) is the reference heart rate obtained from the ECG ground truth signal for the same window *j*. This evaluation method is commonly used in related work [14,15,22,23].

The quality of the signal and the possibility of the measurement are checked in the TDHR algorithm. When the measurement is not possible due to not satisfying Equation (6), i.e., *λ* > *λ_max_* or due to movement detected by the accelerometer affecting all of the periods between the detected peaks, the measurement result is indicated as invalid. The evaluation of the algorithm is performed in two ways: (i) When the result is not available, the last valid result is used; (ii) only valid results are used to calculate the performance metric; and then the percentage of the valid samples is also given. The results of the evaluation, together with the performance of the SpaMa [14], SpaMaPlus [15], Schaeck2017 [23], CNN average, and CNN ensemble [15] algorithms are presented in Table 1. The TDHR algorithm was evaluated for several lengths of the sliding window: *N* = 1024 (32 s), *N* = 512 (16 s), *N* = 256 (8 s), and *N* = 128 (4 s).

Table 2 presents the comparison of the computational cost of several algorithms. For the TDHR algorithm, the number of operations per second was estimated from a manual analysis of the C code as the number of arithmetic operations needed to obtain a single heart-rate result. The TDHR algorithm requires only a few parameters, as opposed to the CNN algorithms, but it needs storage memory for calculating the LMS array; the size of this memory depends on the window length, *N*.

As can be seen from Table 1 and Table 2, the performance of the proposed TDHR algorithm is similar to *SpaMa*, *SpaMaPlus*, and *Schaeck2017*, while it is worse than *CNN average*, *CNN ensemble*, and *CNN constrained*. The computational costs of *SpaMa*, *SpaMaPlus*, and *Schaeck2017* are not known, but they can be high, as those algorithms require the calculation of the power spectral density and the analysis of the PPG spectrum. The CNN-based algorithms, even *CNN constrained*, require larger computational costs than most versions of the TDHR algorithm. The proposed algorithm uses the mechanism of removing the time periods with body motion registered by the accelerometer, so there can be gaps between valid measurements in the case of long-lasting motions. The presented results in Table 1 and Table 2 can help to select *N* to achieve a compromise between the accuracy and computational cost.

## 6. Implementation

**Figure 8:**
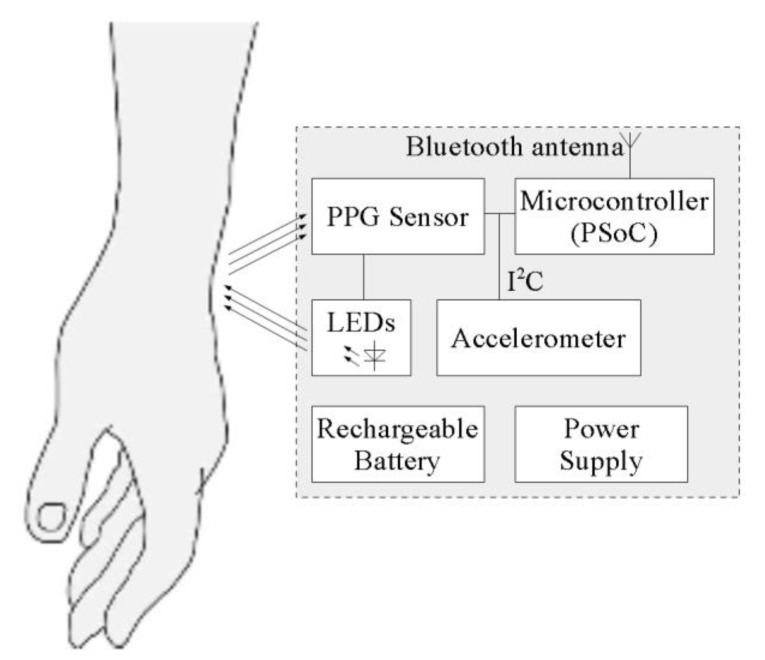
Block diagram of the device implementing the algorithm for measuring the heart rate.

The proposed algorithm was implemented in low-power wearable hardware and tested. The hardware consists of the main board, power supply PCB, with external coil for wireless inductive charging, and a LiPo battery of capacity 110 mAh. A block diagram of the hardware is presented in Figure 8. As the processing unit, a PSoC6 microcontroller from Cypress was used. This is an ultra-low-power microcontroller with dual processor architecture: Arm Cortex M4 and M0+ cores. A BH1790GLC optical sensor from Rohm detects the PPG signal and also drives the four green 527 nm LEDs, equally placed in a circle of diameter of 6 mm, with the optical sensor placed in the center. The detector detects the light emitted by the LEDS and reflected from the patient’s skin. An LSM6DSL accelerometer from STMicroelectronics is mounted on the same board as the optical sensor. The optical sensor and the accelerometer are connected to the microcontroller via an I^2^C bus, used for configuration and data transfer.

The software for heart rate measurements runs on the Cortex M4 processing core of the PSoC6 microcontroller. The algorithm presented in this paper was written in C as two tasks running on the FreeRTOS operating system: The first task constantly reads data from the sensors, realizes filtering, and buffers data. The second task starts periodically and executes the peak detection algorithm, using data from the buffer. The calculated heart rate is sent wirelessly, using the Bluetooth Low Energy protocol. For this purpose, a built-in BLE transceiver in PSoC6 device was used. According to the BLE nomenclature, in the proposed solution, the BLE transceiver block was configured to perform a peripheral (a device constrained in resources such as energy and computing power) and server role (a device working as a data source and sending that data to the remote master device). As a data format, standard BLE Heart Rate Profile was used. The values of the measured heart rate are transmitted periodically; the user can receive the transmitted values by connecting any Bluetooth receiver compatible with Heart Rate Profile.

The PPG signal is sampled with 14-bit resolution and with frequency *f_s_* = 32 Hz, using a buffer of *N* = 128… 1024 samples, which enables data from 4 to 32 s to be analyzed, depending on the selected value of *N*, compromising accuracy versus computational cost and memory usage.

**Figure 9:**
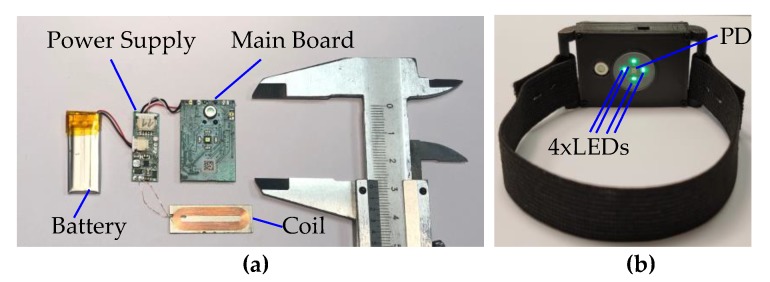
The prototype implementation of a wrist sensor for heart rate measurements with the implemented TDHR algorithm: (**a**) the picture of the internal modules and parts; (**b**) the picture of the final device with strap. The main board has the dimensions of 20 × 30 mm.

The prototype device was built and installed inside a custom-made 3D-printed case with a rubber strap, as shown in Figure 9. The size of the printed case (without the rubber strap) is 11.7 × 26 × 46 mm. The cost of the components, including the battery, was about $60, at retail prices, for 15 pieces. The proposed implementation was capable of continuous measuring of the heart rate for more than 24 h.

## 7. Discussion and Conclusions

Nowadays, there are many smart watches on the market that are capable of measuring the heart rate. The details regarding the optical part of the measurement, such as the number of sensors or the wavelengths of the LEDs used during measurement, are often revealed. However, the details regarding the algorithms used are not available. Most of the publications focus on measuring the accuracy of the popular devices. In [24], the authors measured the performance of Apple’s iWatch Apple Watch Sport 42 mm (first generation), during cardiopulmonary exercise test (CPET). They observed MAE from 6.34 to 7.55. It is difficult to exactly compare this to our results, as we use different and longer test conditions. In fact, the details of the PPG algorithms can only be found in the scientific publications, where the authors try to increase the accuracy of the measurement by using novel ideas and powerful techniques.

In this paper, a time-domain algorithm for the real-time detection of the heart rate was presented. The algorithm is aimed at wearable, resource-constrained devices, where battery capacity, processor speed and memory size are constrained. The algorithm processes raw data in a time-domain and requires only a few parameters. The approach is simple, the proposed algorithm has a reasonable accuracy, and it can be implemented in a typical (not DSP) microcontroller.

The algorithm consists of a two-stage input-signal-conditioning block with a limiter, the peak detection block, and period calculation block. The two-stage input conditioning block is built out of two digital bandpass filters, where the first filter has been modified to process data nonlinearly, to provide fast recovery after large signal transients. The use of band-pass filters is very simple to implement; it appeared sufficient and very effective for conditioning the signal; therefore, other methods such as wavelet-based baseline removal would not need to be considered.

The peak detection block enables operation at significantly lower processing and implementation costs, compared to the original AMPD algorithm. The period calculation block uses the median to calculate the heart rate based on the time differences between the peaks, with the use of an accelerometer to exclude the time periods affected by body movement.

In this study, most of the parameters were set experimentally, as we targeted on a simple and economical hardware implementation. It would be interesting to provide a method of automatic and dynamic adjustment of the parameters to further reduce the computational cost, basing on the input signal quality and the movement readouts. This will be a topic of our further research.

The proposed algorithm was evaluated in several variants, for different sliding window lengths, *N*, providing the possibility to compromise the accuracy versus lower operational costs. The authors mainly used *N* = 1024, which seems to be a good compromise between the calculation cost and the accuracy. The proposed algorithm was compared to the other algorithms from the literature. The achieved accuracy is comparable to the other algorithms at smaller computational costs. The proposed solution was also implemented in a low-power wrist-wearable device.

## Figures and Tables

**Table 1:** Comparison of the accuracy of the presented algorithm to the algorithms from the literature on the large PPG-DaLia dataset as MAE (bpm). The proposed TDHR algorithm was tested in several versions, with various numbers of analyzed samples *N* (for all other algorithms *N* = 256), sampled with a frequency of 32 Hz, with a *BPM_min_* = 40 bpm. The measurements were done every 2 s. For the TDHR algorithm, the accuracy was also calculated for valid measurements, and then the percentage of valid measurements was also given.

	S1	S2	S3	S4	S5	S6	S7	S8	S9	S10	S11	S12	S13	S14	S15	All
SpaMa [14]	11.86	14.75	9.53	17.2	39.28	16.78	15.88	15.2	17.19	9.08	21.63	12.63	9.5	10.73	12.23	15.56 ± 7.5
SpaMaPlus [15]	8.86	9.67	6.4	14.1	24.06	11.34	6.31	11.25	16.04	6.17	15.15	12.03	8.5	7.76	8.29	11.06 ± 4.8
Schaeck 2017 [23]	33.05	27.81	18.49	28.82	12.64	8.72	20.65	21.75	22.25	12.6	21.05	22.74	27.71	12.05	16.4	20.45 ± 7.1
CNN average [15]	8.45	7.92	5.96	7.86	18.97	13.55	5.16	11.49	10.65	6.07	9.87	9.95	5.25	5.85	5.25	8.82 ± 3.8
CNN ensemble [15]	7.73	6.74	4.03	5.9	18.51	12.88	3.91	10.87	8.79	4.03	9.22	9.35	4.29	4.37	4.17	7.65 ± 4.2
TDHR *N* = 1024	8.10	7.98	10.51	11.82	20.60	12.11	7.62	11.71	14.79	4.92	20.05	8.76	7.88	9.15	8.45	10.96 ± 4.49
TDHR *N* = 1024 only valid measurements (%)	5.32 74%	4.99 86%	6.47 71%	6.57 72%	16.26 43%	8.29 55%	5.40 80%	10.28 80%	6.93 64%	3.01 81%	9.17 66%	5.22 61%	4.70 83%	4.84 79%	4.12 75%	6.77 ± 3.26 72%
TDHR *N* = 512	8.61	7.49	11.55	11.93	20.79	14.24	7.96	11.91	14.95	5.83	19.60	8.86	8.22	9.10	8.66	11.31 ± 4.41
TDHR *N* = 512 only valid measurements (%)	6.00 74%	5.60 86%	7.72 73%	7.55 71%	16.05 58%	9.62 65%	6.01 81%	10.50 79%	8.79 65%	3.71 80%	12.15 71%	5.65 62%	5.27 84%	5.34 78%	4.82 75%	7.65 ± 3.30 74%
TDHR *N* = 256	11.08	10.84	11.63	14.06	21.67	15.63	8.86	13.30	15.12	7.27	21.08	10.49	9.54	10.24	9.04	12.66 ± 4.25
TDHR *N* = 256 only valid measurements (%)	8.40 74%	8.21 86%	9.40 75%	10.10 73%	17.02 69%	12.85 72%	7.57 83%	12.04 78%	11.75 68%	5.14 79%	13.86 74%	7.73 62%	7.33 84%	7.45 81%	6.63 74%	9.70 ± 3.21 76%
TDHR *N* = 128	13.13	13.57	13.11	15.59	25.55	18.05	10.25	16.19	19.23	10.32	19.39	12.64	11.63	12.33	12.80	14.92 ± 4.16
TDHR *N* = 128 only valid measurements (%)	12.71 80%	12.25 89%	11.93 82%	14.05 80%	20.84 82%	16.44 79%	9.53 86%	15.65 83%	17.53 78%	8.75 85%	17.13 82%	12.02 72%	10.29 86%	10.66 85%	10.73 80%	13.37 ± 3.46 82%

**Table 2:** Comparison of the performance versus computational cost of the TDHR algorithm and the algorithms from the literature. The computational cost of the TDHR algorithm was calculated as the number of arithmetical operations and the number of memory bytes needed for algorithm realization in a microcontroller.

Algorithm	Performance Mean MAE ± STD (% = Only Valid Measurements)	Computational Cost	
		Number of Parameters/Memory Bytes	Operations Per Second
CNN average	8.82 ± 3.8	8.5 M	34.5 M
CNN ensemble	7.65 ± 4.2	60 M	240 M
CNN constrained	9.99 ± 5.9	26 K	190 K
TDHR *N* = 1024	10.96 ± 4.49	66 k	2.4 M
TDHR *N* = 1024	6.77 ± 3.26 (72%)		
TDHR *N* = 512	11.31 ± 4.41	16 k	598 k
TDHR *N* = 512	7.65 ± 3.30 (74%)		
TDHR *N* = 256	12.66 ± 4.25	4 k	152 k
TDHR *N* = 256	9.70 ± 3.21 (76%)		
TDHR *N* = 128	14.92 ± 4.16	1 k	40 k
TDHR *N* = 128	13.37 ± 3.46(82%)		
